# Myocardial characterisation in Becker muscular dystrophy using T1 and T2 mapping

**DOI:** 10.1186/1532-429X-17-S1-P267

**Published:** 2015-02-03

**Authors:** Marcel Toussaint, Raymond Gilles, Andreas Greiser, Benjamin Marty, Pierre Carlier, Karim Wahbi

**Affiliations:** NMR, Hôpital Pitié-Salpêtrière-Institut de myologie, Paris, France; Imaging & Therapy Division, Siemens Healthcare, Erlangen, Germany; MIRCen, I2BM, CEA, Paris, France; Hôpital Pitié-Salpêtrière-Institut de myologie, Paris, France; CHWAPI, Tournai, Belgium

## Background

Becker muscular dystrophy (BMD) is a severe genetic disease caused by an X-linked recessive mutation leading to reduced/abnormal dystrophin, resulting in a peripheral myopathy and a cardiomyopathy (CM). The purpose of this study is to evaluate the myocardial structure and function in BMD.

## Methods

From April 2012 to May 2014, 84 consecutive patients (age=38,6 ± 13,6 years) with Becker muscular dystrophy (BMD) and 12 control subjects underwent cardiac magnetic imaging (MRI) on a 3-T clinical scanner (Magnetom Trio Tim®, Siemens Healthcare). T1 maps and apparent extracellular volume fraction (appECV) pre- and post-Gd-DOTA injection was performed with a prototype modified Look-Locker sequence (MOLLI, 3, 3 and 5 image acquisitions). T2 maps were generated with a T2 prepared TrueFISP sequence acquired with 3 preparation times (0, 25 and 55 ms). Late Gadolinium enhancement (LGE), T1, T2, appECV were assessed according to the classification of the AHA segmentation.

## Results

Mean left ventricular (LV) ejection fraction was 61±12% (range: 23-87 %), LV end-diastolic volume was 68±26 ml/m2 (range: 32-185 ml/m2) and LV mass 76±17 g/m2 (range: 48-122 g/m2). The values of native T1, GdT1, T2 and appECV of the LW l and the septum are summarized in table 1; they were compared using a Student's *t test* for paired data. The abnormality cut-off values of GdT1 and T2 in the control group were respectively 400 ms and 53 ms. Combining both Gd T1 and T2 data (table 2), fibrosis was detected in the lateral wall: (8% segments) and the septum: (2%) (decreased GdT1, normal native T2), inflammation in 12% *vs* 8% (normal T1 and increased T2), both fibrosis and inflammation in 1,2% vs 0.6 % (decreased T1 and increased T2).

**Table 1:** Relaxometry data.

	Lateral Wall	Septum	p
T1 native (ms)	1205.1 ± 94,1	1183.5 ± 58.2	0.0001
T1 Gd (ms)	506.9 ± 87.2	538.9 ± 87.2	0.0001
T2 (ms)	46.3 ± 5.8	45,0 ± 6.0	0.002
appEVF (%)	36,0 ± 11.3	30.5 ± 11.3	0.0001

**Table 2:** Classification of segments according to the relaxometry data.

T1 Gd (ms)	T2 (ms)	SEPTUM (ms)	LATERAL WALL (ms)
> 400	< 53	284	252
> 400	> 53	27	38
< 400	< 53	6	25
< 400	> 53	2	4
Sum		319	319

## Conclusions

The LGE patterns are different in primitive CM and BMD: in primitive CM, LGE is usually mid-parietal and circumferential; in BMD, it is mainly lateral and sub-epicardial. Relaxometry data demonstrate that the main lesions are inflammatory and fibrotic. The coexistence of both inflammation and fibrosis is uncommon in the same segments. T2 mapping is a useful tool to differentiate fibrosis from both fibrosis and edema. Becker cardiomyopathy may be considered as a "chronic active genetic myocarditis". Further clinical and MRI investigations must explore if the relaxometry data may help to predict long-term survival in Becker dystrophy.

## Funding

N/A.

